# Supplementary material for: Characterization of Systemic Disease Development and Paw Inflammation in a Susceptible Mouse Model of Mayaro Virus Infection and Validation Using X-ray Synchrotron Microtomography
Source: Int J Mol Sci. 2023 Mar 2;24(5):4799. doi: 10.3390/ijms24054799 (PMC10003659; doi:10.3390/ijms24054799)
Supplement: Supplementary file 1 [file ijms-24-04799-s001.zip › ijms-2065595-supplementary.pdf]

International Journal of
Molecular Sciences

MDPI

Supplementary Materials

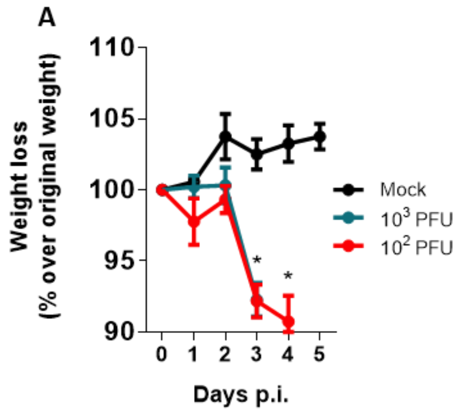

Figure S1 - Peak of disease signs in immunodeficient IFNAR-/- mice occurs on day 3 post-infection. (A) Weight loss throughout infection, an objective and measurable parameter, representing the development of disease signs observed in IFNAR-/- mice inoculated with 10^2 or 10^3 PFU MAYV via intraplantar. Appearance of ruffled fur, conjunctivitis, hunched posture, reduced exploratory behavior and movement are concomitant to development of weight loss. Data presented as mean ± SEM. * p<=0.05

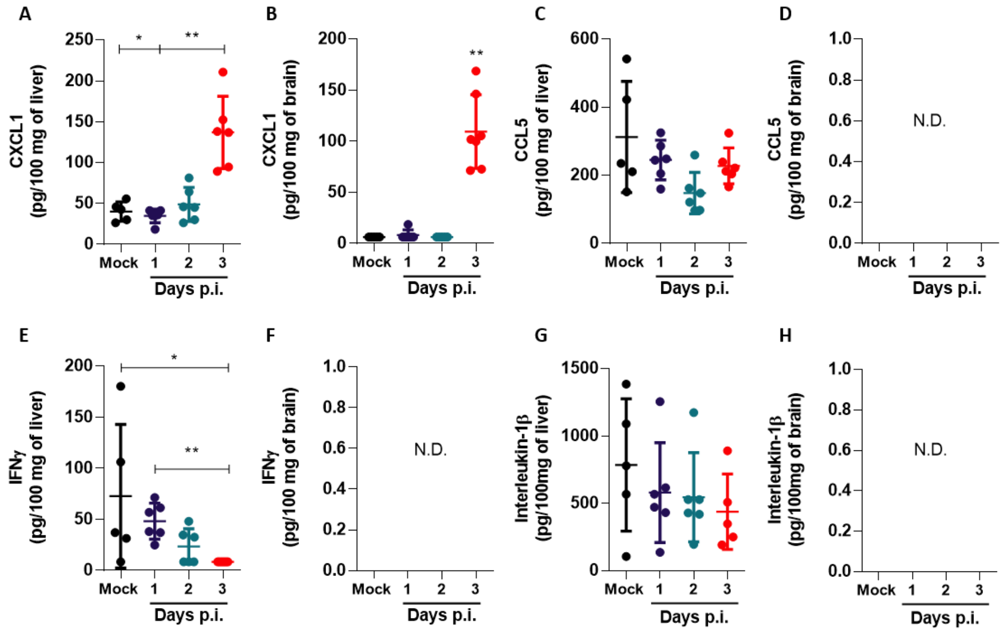

Figure S2 - Production of pro-inflammatory chemokines and cytokines in immunodeficient IFNAR$^{-/-}$ mice upon MAYV footpad inoculation. Levels of (A-B) CXCL1, (C-D) CCL5, (E-F) IFN-γ and (G-H) IL-1β chemokines and cytokines in brains and livers of IFNAR$^{-/-}$ mice inoculated with 10^2 PFU MAYV via intraplantar administration at day 3 p.i.. *N.D. = not detected*. Data presented as mean ± SD. * p<=0.05, **p<=0.01

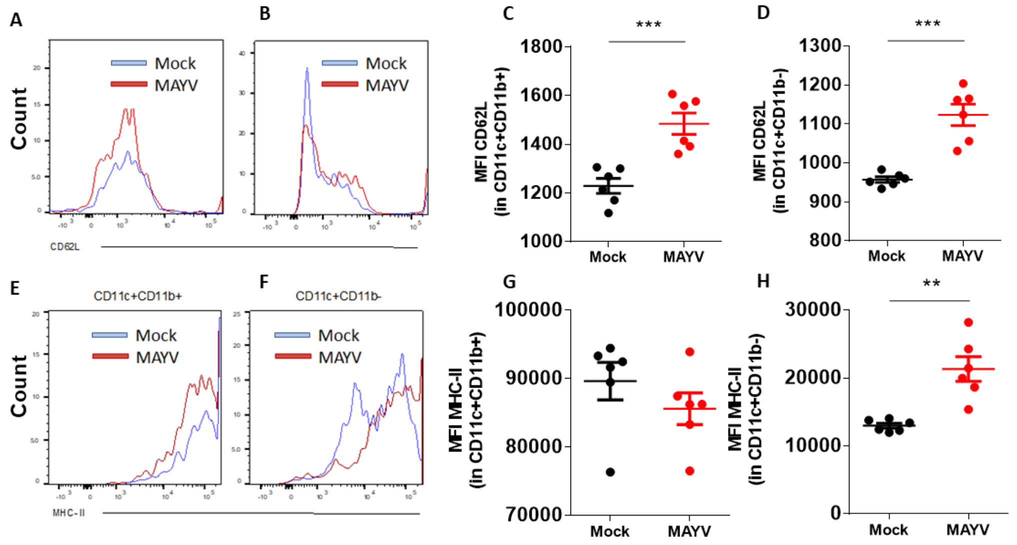

Figure S3 - Dendritic cells show an activated profile and upregulation of surface markers in immunodeficient IFNAR$^{-/-}$ mice upon MAYV footpad inoculation. Spleens of mice infected with 10^2 PFU MAYV via intraplantar route were collected on day 3 p.i. and prepared for flow cytometry for assessment of leukocyte populations and their functional state. (A-B) Histograms of CD62L expression in (A) CD11b$^+$ and (B) CD11b$^-$ DCs. (C-D) Medium fluorescence intensity (MFI) of CD62L in (C) CD11b$^+$ and (D) CD11b$^-$ DCs. (E-F) Histograms of MHC-II expression in (E) CD11b$^+$ and (F) CD11b$^-$ DCs. (G-H) MFI of MHC-II in (G) CD11b$^+$ and (H) CD11b$^-$ DCs. Data presented as mean ± SEM. **p<=0.01, ***p<=0.001

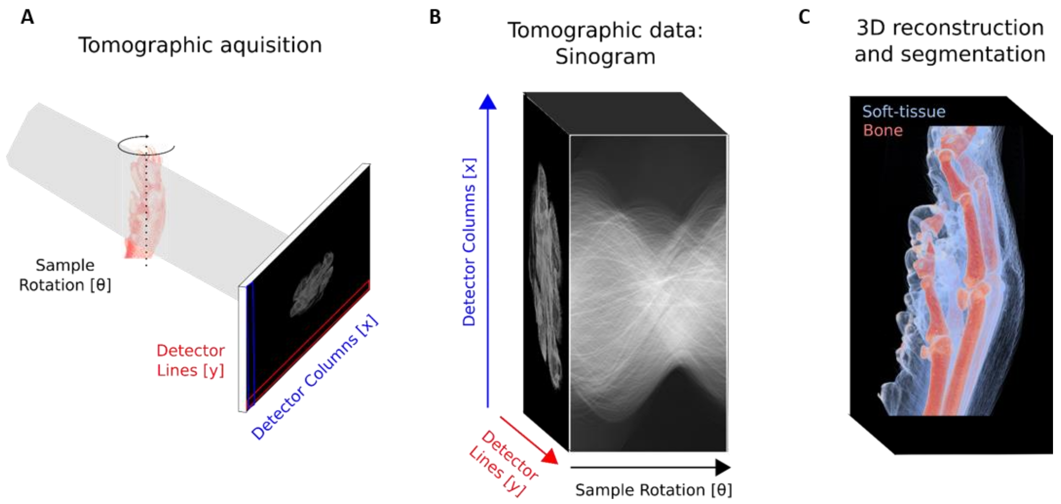

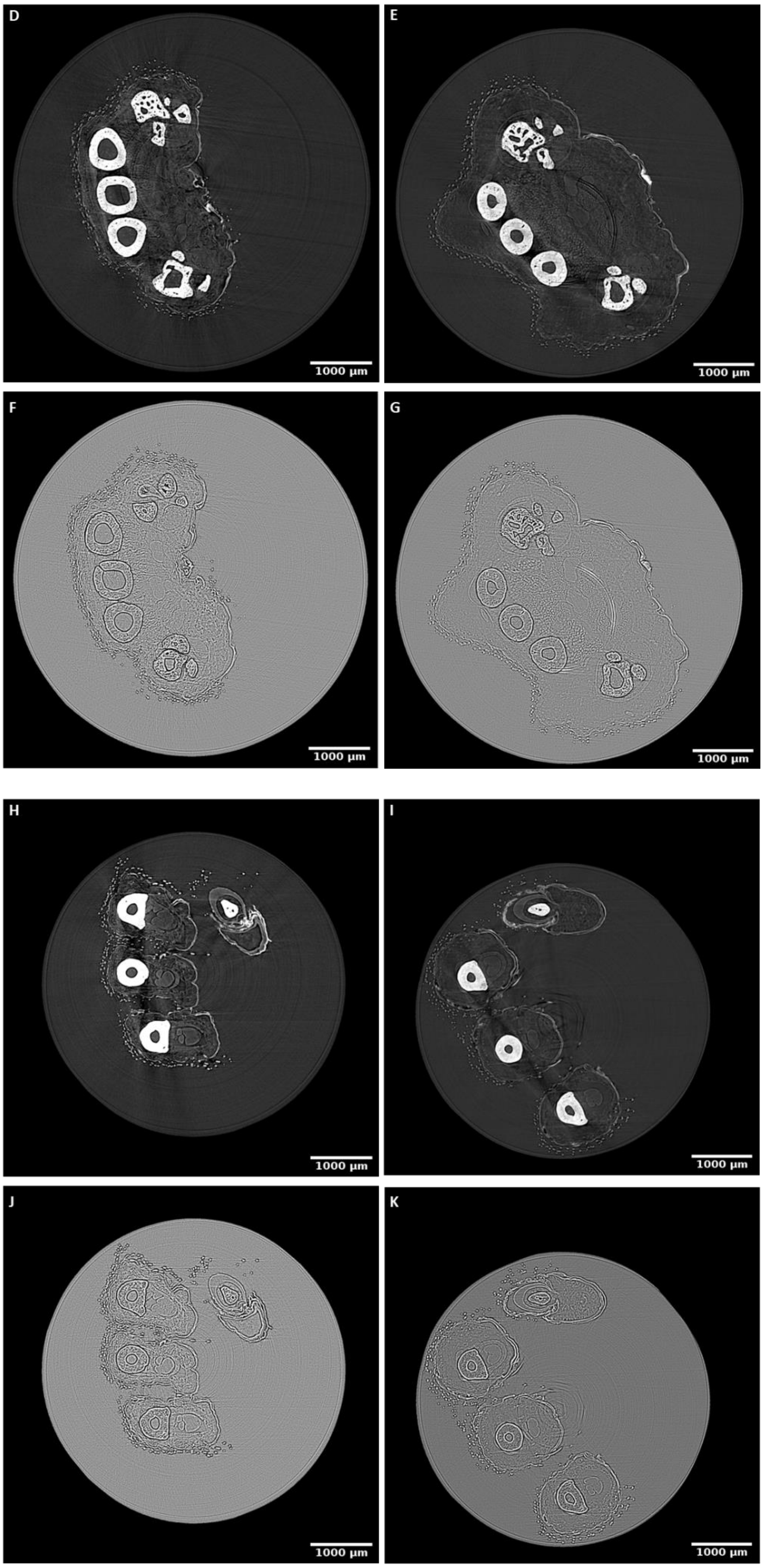
D
1000 µm
E
1000 µm
F
1000 µm
G
1000 µm
H
1000 µm
I
1000 µm
J
1000 µm
K
1000 µm

Figure S4 - Semi-automated microtomography method to assess paw inflammation in MAYV-infected mice and virtual histology obtained from the 3D reconstruction. Schematic representation of the microtomography method developed for assessment of paw inflammation in IFNAR$^{-/-}$ mice inoculated with 10^1 PFU MAYV via intraplantar injection. (A) Hind paws of mock- and MAYV-infected mice were collected and mounted at a rotation stage. During acquisition all parameters were kept the same. Optimization on individual samples were discarded in favor of data regularity. (B) Sinogram reconstruction was performed using filter back-projection (FBP) due to its robustness. (C) 3D structure data of the paw, the semi-automated segmentation of tissues and quantitative analysis with high reproducibility. (D), (F), (H) and (J) are slices from the same mock paw. (E), (G), (I) and (K) are from the same MAYV-infected paw. For better visualization of the soft-tissue, images (F), (G), (J) and (K) were filtered on FBP using a tukey window. Images (D), (E), (H) and (I) were parzen filtered on FBP. This las reconstruction was used on the semi-automatic segmentation procedure.
